# Can mass education and a television campaign change the attitudes towards cardiopulmonary resuscitation in a rural community?

**DOI:** 10.1186/1757-7241-21-39

**Published:** 2013-05-15

**Authors:** Anne Møller Nielsen, Dan Lou Isbye, Freddy Knudsen Lippert, Lars Simon Rasmussen

**Affiliations:** 1 Department of Anaesthesia, Centre of Head and Orthopaedics, Copenhagen University Hospital, Rigshospitalet, Blegdamsvej 9, Copenhagen, 2100, Denmark; 2 Emergency Medicine and Emergency Medical Services, Head Office, The Capital Region of Denmark, Telegrafvej 5, Ballerup, DK-2750, Denmark

**Keywords:** Out of hospital cardiac arrest, Cardiopulmonary resuscitation, Attitudes, Mass media, Bystander basic life support

## Abstract

**Background:**

Survival after out-of-hospital cardiac arrest (OHCA) is improved when bystanders provide Basic Life Support (BLS). However, bystander BLS does not occur frequently. The aim of this study was to assess the effects on attitudes regarding different aspects of resuscitation of a one-year targeted media campaign and widespread education in a rural Danish community. Specifically, we investigated if the proportion willing to provide BLS and deploy an automated external defibrillator (AED) increased.

**Methods:**

BLS and AED courses were offered and the local television station had broadcasts about resuscitation in this study community. A telephone enquiry assessed the attitudes towards different aspects of resuscitation among randomly selected citizens before (2008) and after the project (2009).

**Results:**

For responses from 2008 (n = 824) to 2009 (n = 815), there was a significant increase in the proportions who had participated in a BLS course within the past 5 years, from 34% to 49% (p = 0.0001), the number willing to use an AED on a stranger (p < 0.0001), confident at providing chest compressions (p = 0.03), and confident at providing mouth-to-mouth ventilations (MMV) (p = 0.048). There was no significant change in the proportions willing to provide chest compressions (p = 0.15), MMV (p = 0.23) or confident at recognizing a cardiac arrest (p = 0.09). The most frequently reported reason for not being willing to provide chest compressions, MMV and use an AED was insecurity about how to perform the task.

**Conclusion:**

A targeted media campaign and widespread education can significantly increase the willingness to use an AED, and the confidence in providing chest compressions and MMV. The willingness to provide chest compressions and MMV may be less influenced by a targeted campaign.

## Background

Survival after out-of-hospital cardiac arrest (OHCA) depends on the four links in the chain of survival: recognition of the event, cardiopulmonary resuscitation (CPR), defibrillation and post resuscitation care [1]. Bystanders play an important role since early basic life support (BLS) can increase the survival chances by 2–3 times [2,3] and the additional deployment of an Automated External Defibrillator (AED) can yield survival rates as high as 49-75% [4,5]. Unfortunately, bystander BLS is performed in only approximately one third of time when bystanders with CPR knowledge are present at OCHA [6,7] though with regional variations [8,9]. Rates of bystander BLS have been reported from 1% [10] to 74% [11]. Bystanders deploy AEDs even less often.

Multiple studies have examined the willingness to act when faced with an OHCA, and commonly cited reasons for reluctance are fear of harming the victim, fear of incorrect BLS performance, physical inability and concerns for liability and transmission of infectious diseases. The relative importance of the different reasons for reluctance varies between studies and countries [12-17].

On the Danish island of Bornholm, in 2004, 22% of witnessed OHCA patients received bystander BLS and none survived an OHCA during the period 2001–2003 [18]. A multi-faceted approach was designed to improve this situation. As a part of this process, we wanted to identify specific barriers preventing bystander interventions, thereby enabling us to target our intervention towards these barriers. The aim of this study was to assess the effects of a one-year targeted media campaign and widespread education in a rural Danish community. Specifically, we investigated if the proportion of community members willing to provide BLS and deploy an AED increased after this education campaign.

## Methods

The data collection was a telephone enquiry conducted by a professional opinion research institute (Jysk Analyse A/S), using a computer-assisted telephone interview (CATI). According to Danish law, approval from the Ethics Committee was not required for this study.

### Sample and data collection

The questionnaire was conducted on the Danish island of Bornholm, where the intervention also took place. Bornholm is an island of 588 km^2^ with a year-round population of 42,000, and 600,000 summer tourist visitors each year. From September 2008, 10,000 24-min DVD-based BLS self-training kits (MiniAnne, Laerdal Medical, Stavanger, Norway) were presented as a donation from a private foundation, TrygFonden (http://www.trygfonden.dk) to the Bornholm year-round community. The training kit consists of a simple, personal resuscitation mannequin together with a DVD with BLS instructions. Traditional 4-hour BLS/AED courses were offered at a modest price. The local television station presented broadcasts about resuscitation, including interviews with bystanders, how to use an AED, and how to sign up for the courses. The television campaign was broadcast in the daily local news program. The Emergency Medical Services recorded data on bystander BLS and AED deployment prospectively.

Using a database with both mobile- and land-line telephone numbers, randomly selected phone numbers were called up to 8 times if the telephone was not answered. Inclusion criteria were age above 15 years and permanent resident on Bornholm. The study was performed from September 23rd to 25th 2008, and repeated from September 21st to 24th 2009. The results were afterwards matched according to gender and age with the population on Bornholm. Parts of these results are included in a separate publication [11].

### Questionnaire

The investigators constructed the questions. The first part contained questions relating to baseline characteristics of the respondent. The final part comprised questions about attitudes regarding different aspects of resuscitation and included a hypothetical rescue scenario where the respondents willingness to provide chest compressions, mouth-to-mouth ventilations (MMV) and deploy an AED were assessed from a five-point rating scale; ‘definitely’; ’likely’; ‘unlikely’; ‘definitely not’, and ‘don’t know’. Those unwilling to act (those answering ‘unlikely’ and ‘definitely not’) were asked why they would not, and predefined reasons were provided along with an ’other (please comment)’ option. There was also a question section about the respondent’s self-efficacy in various resuscitation skills. In 2008, there were maximum 17 questions and in 2009 maximum 18. The specific questions can be seen in Tables 1, 2, 3 and 4.

**Table 1:** Baseline characteristics of the participants

	**2008 (N = 824)**	**2009 (N = 815)**
**Age**		
**−15-29 years**	99 (12)	73 (9)
**−30-39**	124 (15)	106 (13)
**−40-49**	165 (20)	155 (19)
**−50-59**	165 (20)	163 (20)
**−60-69**	157 (19)	187 (23)
**- ≥ 70**	115 (14)	130 (16)
**Sex (male)**	379 (46)	357 (44)
Participated in a BLS course within 5 years (yes)*	280 (34)	399 (49)

The numbers are reported as N (%) of respondents. *P = 0.0001, Fishers test.

**Table 2:** Attitudes towards different aspects of resuscitation performed as a telephone enquiry on the Danish island of Bornholm in 2008 and 2009

	**2008 (N = 824)**	**2009(N = 815)**	
**Willingness to provide chest compressions to a stranger**			
**-Definitely**	486 (59)	513 (63)	
**-Likely**	214 (26)	196 (24)	
**-Unlikely**	58 (7)	41 (5)	
**-Definitely not**	58 (7)	49 (6)	
**-Don’t know**	16 (2)	24 (3)	P = 0.15
**Willingness to provide mouth-to-mouth ventilation to a stranger**			
**-Definitely**	478 (58)	481 (59)	
**-Likely**	321 (28)	236 (29)	
**-Unlikely**	58 (7)	41 (5)	
**-Definitely not**	41 (5)	49 (6)	
**-Don’t know**	25 (3)	16 (2)	P = 0.23
**Willingness to use an AED on a stranger**			
**-Definitely**	363 (44)	530 (65)	
**-Likely**	157 (19)	139 (17)	
**-Unlikely**	157 (19)	49 (6)	
**-Definitely not**	115 (14)	65 (8)	
**-Don’t know**	41 (5)	24 (3)	P < 0.0001
**Confident at recognizing an OHCA?**			
**-Definitely**	247 (30)	220 (27)	
**-Likely**	338 (41)	391 (48)	
**-Unlikely**	140 (17)	114 (14)	
**-Definitely not**	58 (7)	57 (7)	
**-Don’t know**	33 (4)	33 (4)	P = 0.09
**Confident at providing chest compressions?**			
**-Definitely**	288 (35)	334 (41)	
**-Likely**	305 (37)	302 (37)	
**-Unlikely**	115 (14)	82 (10)	
**-Definitely not**	99 (12)	82 (10)	
**-Don’t know**	16 (2)	16 (2)	P = 0.03
**Confident at providing mouth-to-mouth ventilation?**			
**-Definitely**	371 (45)	416 (51)	
**-Likely**	280 (34)	245 (30)	
**-Unlikely**	82 10	65 (8)	
**-Definitely not**	66 (8)	65 (8)	
**-Don’t know**	8 (1)	16 (2)	P = 0.048
**What is the survival rate to hospital discharge after OHCA?**			
**−0**	33 (4)	41 (5)	
**- ≤ 10%**	214 (26)	179 (21)	
**−11-20%**	99 (12)	90 (11)	
**−21-30%**	107 (13)	98 (12)	
**−31-40%**	58 (7)	41 (5)	
**−41-50%**	99 (12)	106 (13)	
**- ≥ 50%**	82 (10)	90 (11)	
**-Don’t know**	132 (16)	179 (22)	P = 0.01
**What is the survival rate to hospital discharge after OHCA if BLS is provided immediately?**			
**−0**	0	0	
**- ≤ 10%**	33 (4)	16 (2)	
**−11-20%**	49 (6)	24 (3)	
**−21-30%**	49 (6)	33 (4)	
**−31-40%**	25 (3)	24 (3)	
**−41-50%**	115 (14)	139 (17)	
**- ≥ 50%**	445 (54)	456 (56)	
**-Don’t know**	107 (13)	122 (15)	P = 0.002
**What is the survival rate to hospital discharge after OHCA if BLS and AED use is provided immediately?**			
**- ≤ 10%**	16 (2)	8 (1)	
**−11-20%**	25 (3)	8 (1)	
**−21-30%**	33 (4)	16 (2)	
**−31-40%**	25 (3)	16 (2)	
**−41-50%**	49 (6)	57 (7)	
**- ≥ 50%**	569 (69)	579 (71)	
**-Don’t know**	115 (14)	122 (15)	P = 0.005
**What is the prognosis after OHCA survival?**			
**-Very poor (nearly all die or suffer severe brain injury)**	66 (8)	57 (7)	
**-Generally poor (the majority die or suffer severe brain injury)**	165 (20)	155 (19)	
**-Neither poor nor good**	181 (22)	147 (18)	
**-Generally good (few disabilities)**	313 (38)	334 (41)	
**-Very good (no disabilities)**	33 (4)	65 (8)	
**-Don’t know**	58 (7)	57 (7)	P = 0.008
**Would you want other laypersons to try to resuscitate you if you suffered OHCA?**			
**-Definitely**	692 (84)	701 (86)	
**-Likely**	58 (7)	57 (7)	
**-Unlikely**	25 (3)	8 (1)	
**-Definitely not**	25 (3)	16 (2)	
**-Don’t know**	33 (4)	24 (3)	P = 0.03

The numbers are reported as N (%) of respondents. Chi-squared test is used for calculation of p-values. AED: Automated External Defibrillator. OHCA: out-of-hospital cardiac arrest. BLS: Basic Life Support.

**Table 3:** Reasons for not being willing to perform chest compressions, mouth-to-mouth ventilations and use an Automated External Defibrillator (AED)

**Reasons for not being willing to…**	**…provide chest compressions**		**…provide mouth-to-mouth ventilations**	
**p-value**	0.82		0.41	
	2008 (N = 114)	2009 (N = 89)	2008 (N = 94)	2009 (N = 90)
**Don’t know how to**	62 (54)	43 (48)	41 (44)	32 (35)
**Afraid of doing harm**	29 (25)	21 (24)	10 (11)	15 (17)
**Don’t think it helps**	1 (1)	1 (1)	5 (5)	3 (3)
**Don’t want to touch stranger**	-	-	9 (10)	5 (5)
**Afraid of transmittable diseases**	-	-	5 (5)	4 (4)
**Other**	21 (18)	22 (25)	23 (24)	28 (31)
**Don’t know**	2 (2)	1 (1)	1 (1)	4 (4)

Numbers are percentages of respondents. Chi-squared test is used for calculation of p-values.

**Table 4:** Attitudes towards different aspects of resuscitation in 2009

**Have you participated in a BLS course within 5 years? (2009)**	**Yes (N = 372)**	**No (N = 441)**	
**Willingness to provide chest compressions to a stranger**			
**-Definitely**	286 (77)	216 (49)	
**-Likely**	78 (21)	119 (27)	
**-Unlikely**	4 (1)	40 (9)	
**-Definitely not**	4 (1)	44 (10)	
**-Don’t know**	4 (1)	22 (5)	P < 0.0001
**Willingness to provide mouth-to-mouth ventilation to a stranger**			
**-Definitely**	253 (68)	216 (49)	
**-Likely**	104 (28)	128 (29)	
**-Unlikely**	7 (2)	31 (7)	
**-Definitely not**	4 (1)	44 (10)	
**-Don’t know**	4 (1)	18 (4)	P < 0.0001
**Willingness to use an automated external defibrillator on a stranger**			
**-Definitely**	283 (76)	247 (56)	
**-Likely**	56 (15)	88 (20)	
**-Unlikely**	19 (5)	35 (8)	
**-Definitely not**	11 (3)	101 (13)	
**-Don’t know**	4 (1)	18 (4)	P < 0.0001

Numbers are percentages of respondents. Chi-squared test is used for calculation of p-values.

### Data analysis

Descriptive statistics were used to characterize the sample and each of the questions. Responses from 2008 and 2009 were independent. Fisher’s test and chi-squared test was used for comparisons between 2008 and 2009 responses. A p-values < 0.05 were considered statistically significant, but to adjust for multiple testing a Bonferroni correction was applied (p < 0.003). In order to obtain a sample size of 800, which is necessary for a representative selection of the population, we aimed at contacting 1150 citizens at both assessments.

## Results

**Figure 1:**
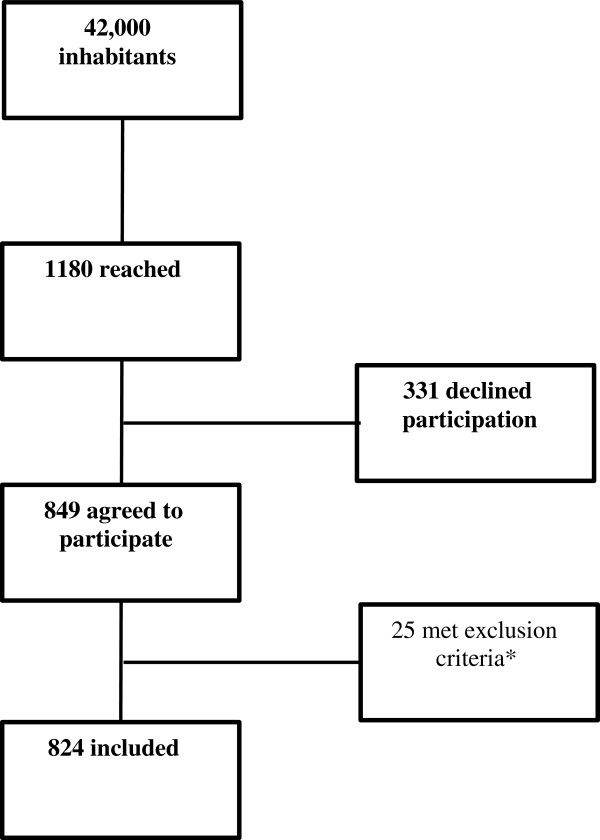
**The flow of the participants in 2008.** *Age below 15 years or non-resident of Bornholm.

**Figure 2:**
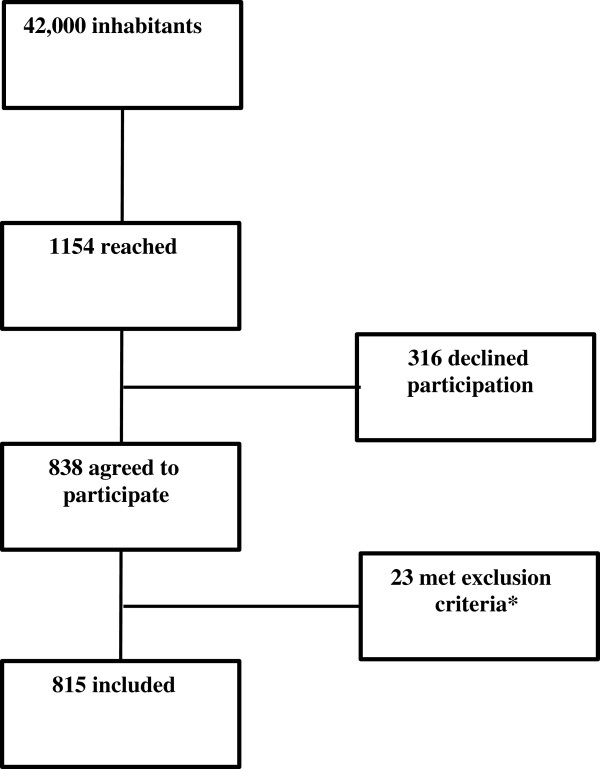
**The flow of the participants in 2009.** *Age below 15 years or non-resident of Bornholm.

In 2008, 1180 residents were contacted, 849 (72%) agreed to participate and 824 (70%) also met the inclusion criteria (age above 15 years and residents of Bornholm), see Figure 1. In 2009, 1154 were contacted, 838 (73%) agreed to participate and 815 (71%) also fulfilled inclusion criteria (Figure 2). When compared to the entire population of Bornholm [19], there was no difference in gender or age for either group. There was a significant increase (from 34% to 49%, p = 0.0001, 2008 and 2009 respectively) in the proportion who had participated in a BLS course within the past 5 years (Table 1).

Among those reporting in 2009, 75% said that had participated in a course within the past 12 months. These courses were the 24-min self-instruction DVD based MiniAnne course (Laerdal, Stavanger, Norway) in 32% of the cases, a 4-hour BLS/AED course in 36% of the cases, and other BLS courses in 32% of the cases. Also for 2009, there was a significant increase compared to 2008 in the proportions that were willing to use an AED on a stranger, that were confident at providing chest compressions and confident at providing MMV. As far as reported perceptions, the anticipated survival rates for OHCA to hospital discharge were expected to be higher if BLS was provided immediately and/or an AED was used and there was a significant increase in the anticipated survival rate in 2009. Likewise the perception of expected prognosis after OHCA was thought to be better if BLS was performed and the proportion who would want to be resuscitated by a layperson also increased significantly (Table 2).

There was no significant change from 2008 to 2009 in the proportions of those that reported that they were willing to provide chest compressions or mouth-to-mouth ventilation. Likewise the confidence at recognizing a cardiac arrest remained unchanged (Table 2).

In both 2008 and 2009, the most frequently reported reason for not being willing to provide chest compressions, mouth-to-mouth ventilation and use an AED was insecurity about how to perform the task (Table 3).

In 2008 and 2009, 269 and 112 (respectively) reported that they were not being willing to use an AED for the following reasons: ‘don’t know to operate one’ (77% in 2008, 62% in 2009), ‘don’t know what it is’ (11% vs. 5%), ‘afraid of doing harm’ (6% vs. 9%), and ‘other’ (4% vs. 22%). In 2008, 1% indicated that the reason was either ‘don’t know where to find one’ or ‘don’t believe that it helps’; both these reasons were not reported in 2009. There was a significant change in the weighting of the different reasons from 2008 to 2009 (p < 0.0001), with ‘not knowing what an AED is’ moving from being the second most frequently reported reason in 2008 to only the fourth most frequent reported reason in 2009.

Concerning ‘other’ responses, the most frequently reported reason for not being willing to provide chest compressions was physical inability. Other reasons were panic, and for MMV also dislike of the maneuver. Fear of legal consequences was not mentioned. With regard to the AED, the most frequently reported reason in the ‘other’ section was ‘being too old’.

Those who had participated in a BLS course within the past 5 years had a significantly greater willingness to perform BLS and use an AED (Table 4).

## Discussion

We found that the willingness to use an AED and the confidence in providing chest compressions and MMV increased significantly after an intervention with mass education in BLS and a profound media campaign. The willingness to provide chest compressions and MMV remained unchanged.

The intervention significantly increased the proportion who had participated in a BLS course, and we have reported elsewhere that in the same time period the bystander BLS rate for all OHCA on that island was 41.5% [95% CI 28–57] and 80% [95% CI 54–94] for the witnessed [11]. The increase in bystander rate over time most likely reflects effects of the media and educational intervention, since no other changes in resuscitation publications or routines occurred at the same time; guidelines and the dispatch BLS protocol remained the same and no other community BLS interventions took place.

This apparent paradox, that the behavior changed, but not the attitude, might be related to the detail that the question considered a stranger, since other studies have reported less willingness to provide BLS to a stranger as compared to a family member [12,15,20]. We do not know if the bystanders who commenced BLS were close relatives, but 65% of the OHCA victims who received bystander BLS collapsed in public places, probably indicating that they were strangers. Another explanation could be that the bystanders were trained in BLS, and our study showed that those who had participated in BLS training within the past 5 years had a significantly greater willingness to provide all the skills of resuscitation, compared to those who had not been BLS-trained. This finding confirms some other studies [13,20]. A more straightforward explanation for the paradox could be that when asked “would you provide BLS to a victim of OHCA”, few would decline. Then, as more people are trained and receive input from television, BLS confidence as well as the bystander BLS rate increases. Indeed, a previous study has shown that television public service announcements were associated with increase bystander BLS rate [21].

The proportion willing to use an AED increased significantly after the media and educational intervention, which is in agreement with other studies that have shown that even brief training increases the willingness to use an AED [22-24]. How to operate an AED was shown several times on the local television program, and the number of AEDs increased in the period from 3 to 118. An AED was deployed in 10% [95% CI 3–23] (N = 4) of the OHCAs [11] in a following period. Among those unwilling to deploy an AED, only 6-9% (2008 and 2009) reported fear of doing harm as the main reason. It is surprising that people believed that chest compressions and MMV are more “dangerous”, as 25-24% and 11-17%, respectively, reported fear of doing harm as the reason for not being willing to ‘fire’ an AED. This likely reflects implicit trust in the AED, and it may be thought easier to just “let the machine do what is does” than to be responsible for the consequences of one’s own chest compressions.

In 2008 and 2009, only 30% and 27% (respectively) reported that they were ‘definitely’ confident at recognizing an OHCA, which supports the increased emphasis on starting CPR on anyone who is unconscious and not breathing normally [25]. Most people learned and practiced with the short DVD-based course, and one future question could be if enough attention is paid to the recognition of cardiac arrest. Other studies have reported that 4 months after completing these courses, 38% did not attempt to open the airway, up to 30% did not check for breathing and up to 51% did not shake the patient to check for consciousness [26,27].

Many studies have shown reluctance to perform MMV. In our study, 12% in 2008 and 11% in 2009 indicated reluctance to MMV, which is in accordance with a study from Western Australia [20]. In contrast, a Japanese study found that only 15% of high-school students were willing to perform chest compression and MMV to a stranger [15]. The fear of disease transmission (4-5%) differed from other studies. In Sweden, 94% were afraid of at least some disease transmission during CPR [16], the same for 43% of Norwegian secondary school students [28], but only for 5-6% of Japanese high school students and their teachers perceived risk for disease transmission as a problem [14]. This wide spectrum probably reflects difference in research methodology but also cultural differences. These studies were performed in different settings and with different methodologies. Some of the studies examined attitudes in various circumstances, like providing CPR to a trauma patient, drug abuser or a family member. In our study, we only enquired about performing CPR to a stranger, not further specified. Thus, comparison of results from different studies is not simple.

No responses were noted naming fear of legal consequences as a reason for being reluctant to start BLS in our study, but that was the case for 21.6% of the respondents in a study from Arizona, USA [12]. Again, this might reflect cultural but also legislative differences.

The questions about the estimated survival rate and prognosis show a significant increase from 2008 to 2009, especially with regard to the value of BLS and AED’s. In addition, more people would want to be resuscitated themselves. This likely reflects an effect of the intervention, as there were several television broadcast about the poor survival rate and laypersons ability to change this. Likewise many people who were successfully resuscitated from OHCA appeared on television, telling their story. Expectations regarding the prognosis were surprisingly positive, and especially the value of BLS and AED was considered important. An overwhelming majority wanted to be resuscitated themselves. In that perspective it is surprising that not all people were willing to participate in BLS themselves. It is notable that the vast majority responses express a desire to be resuscitated even when the likelihood of successful resuscitation (because of prolonged time in cardiac arrest) is low. More than 80% of the participants with age above 70 years would ‘definitely’ or ‘likely’ want to be resuscitated (results not shown).

There are limitations with our study design. With regard to the course type, only 32% reported that they had completed the MiniAnne course. This is unexpected, since the MiniAnne course was the most frequently offered course and it is possible that the true number of course completions was higher than reported. The answer alternatives were ‘Yes, the MiniAnne course’, ‘Yes, the AED course’ or ‘Yes, other’. Among the answers in the ‘other’ category it is evident that many courses were indeed the MiniAnne course, thus some participants just did not know the name MiniAnne. Unfortunately, more than 25% declined to participate and one may speculate that these individuals probably would be among those with a negative attitude towards resuscitation, though the proportion of non-responders was the same at both occasions.

Many responses were given in the ‘other’ category, indicating that the predefined reasons were possibly too few. Also, as mentioned before, it would have been helpful to know what the participants actually knew about BLS, together their self reported confidence. It might also have been helpful to know who had been exposed to a real OHCA.

The large sample enabled us to detect many significant differences (Table 2). However, the clinical relevance of some of these findings where the changes in some cases were only few percent, may be unclear. Applying a Bonferroni correction does not change the interpretation that a significant increase was found in the proportion willing to use an AED, but the change in confidence with BLS was not significant after correction.

## Conclusion

In a Danish rural island community, mass education in BLS and a television campaign over one year lead to a significant increase in the willingness to use an AED, and the confidence in providing chest compressions and mouth-to-mouth ventilations. The willingness to provide chest compressions and mouth-to-mouth ventilations remained unchanged.

## Abbreviations

AED: Automated external defibrillator; BLS: Basic life support; CPR: Cardiopulmonary resuscitation; OHCA: Out of hospital cardiac arrest; MMV: Mouth-to-mouth ventilation.

## Competing interests

The authors declare that they have no competing interests. An employee, Rune Holm, of the local television station, TV2/Bornholm, was engaged in the project, among other tasks. There was no financial relationship between the television station and the project. Laerdal’s contribution was solely as the manufacturer.

## Authors’ contributions

All authors made contributions to conception and design and interpretation of data. AMN drafted the manuscript and AMN and LSR performed the statistical analysis. LSR, DLI and FKL have revised the manuscript for important intellectual content. All authors read and approved the final manuscript.
